# COVID-19 mental health helpline: A tool for a rural population

**DOI:** 10.1192/j.eurpsy.2021.764

**Published:** 2021-08-13

**Authors:** B. Jesus, S. Freitas Ramos, M.I. Fonseca Marinho Vaz Soares, J. Martins Correia, D. Cruz E Sousa, S. Caetano

**Affiliations:** 1 Department Of Psychiatry And Mental Health, Local Health Unit of Guarda, Guarda, Portugal; 2 Mental Health, Hospital, Guarda, Portugal; 3 Psychiatry And Mental Health, Hospital, Guarda, Portugal

**Keywords:** mental health, COVID-19, Helpline

## Abstract

**Introduction:**

Coronavirus disease 2019 (COVID-19) pandemic has had a negative impact for mental health. ULS-Guarda in cooperation with Portugal National Health Service, provided the population of the district of Guarda with a mental health helpline (MHHL).

**Objectives:**

Provide a descriptive data analysis of the MHHL calls received between April 1^st^ and September 20^th^ of 2020.

**Methods:**

The data was obtained through the filling out of questionnaires. It included fields for gender, age, the type of service provided, relation to COVID-19, symptoms displayed and the number calls made per patient. For the statistical analysis, Microsoft Excel ^TM^ was utilized.

**Results:**

MHHL received 191 calls. The largest volume was received during April, which saw 116 instances of patients seeking the MHHL. The number of calls then tapered progressively throughout the following months. The services provided were split between psychiatric assistance, psychologic assistance, and the renovation of medical prescriptions, in 44%, 31% and 19% of the cases, respectively. The 101 patients who resorted to the MHHL were unevenly distributed in gender, being 74 female and 27 male individuals. Their ages were mostly between 50 and 69 years old. The most common symptoms were anxiety, depressed humor and insomnia, in 35%, 16% and 11% of the cases, respectively.

**Conclusions:**

The largest influx of calls coincides with the home confinement period, and decreased alongside the relaxation of the confinement measures held. The MHHL had enough adherence to warrant consideration of it being an alternative means of healthcare access, especially in situations where physical access to healthcare is restricted.

